# The Development and Content Validation of a Clinical Screening Scale to Identify Attention-Deficit Hyperactivity Disorder Cases Based on the Gender Perspective: An e-Delphi Study

**DOI:** 10.3390/healthcare12131282

**Published:** 2024-06-27

**Authors:** Meritxell Perez-Beltran, Juan Roldán-Merino, Maria Eugenia Russi, Maria Garau Rolandi, Roser Colome Roura, Francisco Sampaio, Marta Domínguez Del Campo, Mariona Farres-Tarafa, Barbara Hurtado Pardos, José Ángel Alda Díez

**Affiliations:** 1 School of Nursing, Campus Sant Joan de Déu de Barcelona-Fundació Privada, Calle Sant Benito Menni 18-20, Sant Boi de Llobregat, 08830 Barcelona, Spain; meritxell.perez@sjd.edu.es (M.P.-B.); mariona.farres@sjd.edu.es (M.F.-T.); barbara.hurtado@sjd.edu.es (B.H.P.); 2 Facultat de Psicología, University of Barcelona, Pg. de la Vall d’Hebron, 171, 08035 Barcelona, Spain; 3 Neuropsychologist at Avan Neurology Center, Carrer Estrella, 10, Sabadell, 08201 Barcelona, Spain; 4 Mental Health, Psychosocial and Complex Nursing Care Research Group (NURSEARCH), University of Barcelona, Gran Via de les Corts Catalanes, 585, 08007 Barcelona, Spain; 5 Neuropediatrician in the Pediatric Neurology Service, Sant Joan de Déu Hospital, Pg. de Sant Joan de Déu, 2, 08950 Barcelona, Spain; mariaeugenia.russi@sjd.es; 6 Neurology Service and in the Learning Disorders Unit (UTAE), Sant Joan de Deu Hospital, 08950 Barcelona, Spain; maria.garau@sjd.es (M.G.R.); roser.colome@sjd.es (R.C.R.); 7 Psychology and Neurotherapy Centers, Carrer de Gresolet, 14, Sarrià-Sant Gervasi, 08034 Barcelona, Spain; 8 Nursing School of Porto, Rua Dr. António Bernardino de Almeida, 830, 844, 856, 4200-072 Porto, Portugal; franciscosampaio@esenf.pt; 9 CINTESIS@RISE, Nursing School of Porto (ESEP), Rua Dr. Plácido da Costa, s/n, 4200-450 Porto, Portugal; 10 Parc Sanitari Sant Joan de Déu-Research Center, Carrer del Camí Vell de la Colònia, 25, 08830 Barcelona, Spain; marta.dominguez@sjd.edu.es; 11 Child and Adolescent Psychiatry and Psychology Department, Hospital Sant Joan de Déu of Barcelona, Pg. de Sant Joan de Déu, 2, 08950 Barcelona, Spain; joseangel.alda@sjd.es; 12 Children and Adolescent Mental Health Research Group, Institut de Recerca Sant Joan de Déu, Santa Rosa, 08830 Esplugues de Llobregat, Spain

**Keywords:** hyperactivity, inattention, gender, delphi, instrument development

## Abstract

Background: Although many studies analyse gender differences in the clinical expression of Attention-Deficit Hyperactivity Disorder (ADHD) and prevalence studies show that girls with ADHD are underdiagnosed, there are no instruments that are sensitive to the detection of girls with ADHD. Objective: The objective of this study is to develop a self-report early detection instrument for boys and girls with ADHD aged 7 to 16, which includes the gender perspective and is sensitive to the detection of girls with ADHD. Methods: The scale was developed and the items that comprised it were created from the thematic analysis of ADHD and its evaluation in children based on the diagnostic criteria of the DSM-5-TR. A modified e-Delphi method involving a three-round web survey was used to establish a consensus on the content of the scale. Ten experts were recruited to form a professional panel. The panel members were asked to assess the differential symptomatology of ADHD in boys and girls, the dimensions to be evaluated, and the importance of scale items to evaluate the content. Results: A consensus was reached regarding 13 total items distributed in three dimensions: inattention; hyperactivity/impulsivity; and, a third dimension, internalisation, which includes symptoms most present in the expression of ADHD in girls. Conclusions: To the best of our knowledge, the development of this scale using the Delphi method is the first specific scale used for identifying ADHD that also addresses the gender perspective and the differential symptomatology between boys and girls. However, we must proceed to the analysis of psychometric properties, as the scale requires an exhaustive study of its reliability and validity. We can anticipate that this scale will provide relevant and reliable information that can be used for the identification of ADHD in both boys and girls.

## 1. Introduction

Attention-Deficit Hyperactivity Disorder (ADHD) and its existence have been widely documented in the literature, although it has been criticised as a diagnostic category. Its inclusion as a disorder in the Diagnostic and Statistical Manual of Mental Disorders III (DSM-III) in 1983 generated controversy as it was initially defined by the American Psychological Association (APA) in 1980 as a list of generic behaviours. In 1998, the American Medical Association noted that ADHD was one of the most studied disorders in medicine, recognising its significant prevalence and chronic nature as a public health problem [1].

Since then, it has maintained its status as diagnosable and has become one of the most common childhood disorders, prevalent in 8.8% of the world’s population according to a World Health Organisation (WHO) report published in June 2022 [2]. It is present in 3.1% of adolescents aged 10–14 years and 2.4% of those aged 15 years and older [3]. ADHD is one of the most common chronic disorders in childhood, the effects of which can persist into adolescence and adulthood. With neurobiological aetiology, the core symptoms manifest in childhood before the age of 12, and its course is chronic, evolutionarily symptomatic, and has a high rate of heritability [4]. In Spain, the prevalence rate ranges between 5% and 7% in the school population [5]. Many children and adolescents with ADHD face difficulties with regulating their behaviour, which can result in adjustment problems in family, school, and social settings. They often perform below their ability and may experience emotional and behavioural disturbances [6].

Research has found that children diagnosed with ADHD show comorbidity with other disorders; difficulties in interpersonal relationships; and behavioural problems that directly affect their academic performance, family, and relationships in adulthood [7,8,9,10,11,12,13].

In recent years, there have been in-depth analyses on how gender influences ADHD symptomatology, clinical presentation, diagnosis, and treatment [14,15,16,17,18,19]. Several studies indicate that the symptomatic basis of ADHD differs between boys and girls, with a greater tendency in boys towards hyperactive and impulsive symptoms, while in girls, more inattentive and anxious symptomatology is observed [14,20,21,22,23]. This appears to relate to the perceived stereotype for parents and teachers of ADHD, which tends to be associated with the ‘disruptive child’ in line with perceived DSM-IV diagnostic criteria [24,25,26].

Recent scientific literature studies have documented gender differences in psychosocial, cognitive, and psychiatric functioning in boys and girls with ADHD [27,28,29,30,31,32,33,34]. In addition, under-diagnosis (or under-identification) has been observed in girls with ADHD, with studies suggesting a ratio of diagnosis at younger ages ranging from 2:1 to 10:1, with higher ratios in clinical settings [35,36,37,38,39,40,41].

According to the scientific literature, in the assessment of ADHD, instruments to measure symptoms and executive functions are essential; interviews with the patient and family members, as well as self-report scales and questionnaires, are important to understand the intensity of symptoms and their specificities [42,43]. However, many of the instruments used lack a gender perspective, which highlights the need for tools that are sensitive to the symptomatology presented in girls, as demonstrated by the extensive literature [44]. Some of the most widely used and specific scales that are used for the detection and diagnosis of ADHD do not differentiate between boys and girls [14], and existing instruments are analysed according to gender to make them more reliable in their discriminative function [45,46].

This study aims to design an instrument for the early detection of ADHD that considers the gender perspective and is sensitive to the detection of girls with ADHD. This instrument must have sufficient content validity, must be able to differentiate ADHD from other disorders with which it could be confused, and must be sensitive to the symptomatology of ADHD in girls.

## 2. Methods

### 2.1. Study Design

**Figure 1:**
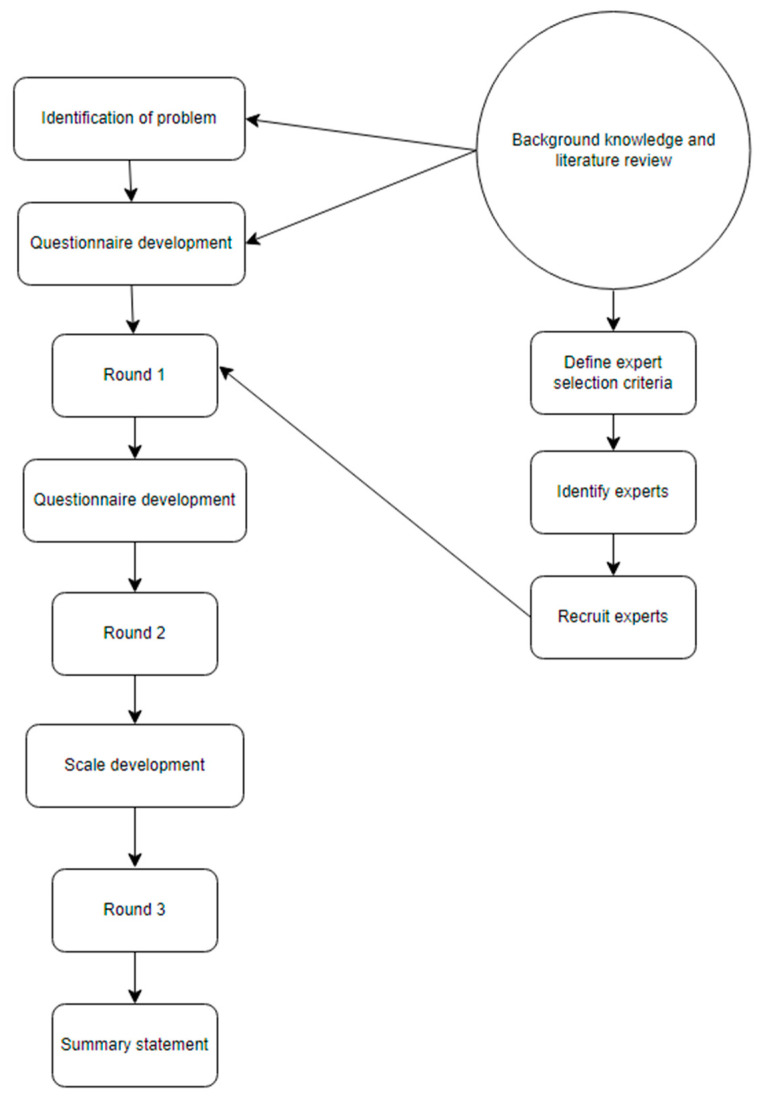
Delphi process flowchart.

For the construction of the ADHD detection scale from a gender perspective, the Delphi technique was used in its electronic form, e-Delphi [47]. Figure 1 shows the procedure followed for the e-Delphi method [48,49].

The Delphi technique has been widely used in health research [47,50,51,52] to converge, and establish a consensus of, opinions in a structured way among a group of experts on different types of issues and topics.

This anonymous process makes it possible to for the group of experts to make accurate assessments about different questions that are posed through multiple rounds of surveys, distributed by e-mail in this case. Anonymity among the expert professionals themselves allows them to express their opinions freely.

In the same way, it prevents influential participants from directing or guiding the answers, and this has an important impact on the results. In this study, the telematic method was used as it allowed the research team to recruit professional experts from different origins and geographical locations. Thus, each professional expert received the information and responded autonomously and individually.

e-Delphi was structured in three rounds; for each round, three specific forms were elaborated and sent telematically to the panel of experts.

In the first round, experts were asked to assess which DSM-5-TR symptoms of inattention and hyperactivity were more frequent in the symptomatological expression of ADHD in girls, and then to identify which symptomatology would be specific only to girls.

The second round consisted of assessing the symptoms identified in the first round specific to ADHD in girls. Given that the symptomatology of inattention and hyperactivity/impulsivity was validated by the DSM-5-TR, the aim of the second round was for the experts to rate the new items that would make up the scale and that would be sensitive to the symptomatology of ADHD in girls.

In the third round, the research team transformed the symptomatology into a broad pull of items to be included in the scale. The items distributed in three dimensions, inattention, hyperactivity/impulsivity, and “internalisation”, made up the first version of the scale that was submitted to experts for feedback. At this point, each item was re-evaluated based on its eligibility to be included or not in the final version and the most suitable format for the scale.

The final proposal of the scale met the criteria of relevance, coherence, and clarity in its formulation to determine the degree of consensus and convergence of opinion.

### 2.2. Theoretical Structure of the Scale

For this study, we used the diagnostic criteria of the Diagnostic and Statistical Manual of Mental Disorders-5-TR (DSM-5-TR) as the basis for developing the scale. These criteria are widely accepted internationally for diagnosing ADHD. A diagnosis of ADHD is based on whether or not a person meets these criteria which describe the main symptoms of the disorder.

Most of the instruments used to diagnose ADHD are based on the perceptions of parents and teachers, but some have been adapted to be completed by the children themselves. After reviewing the literature, we decided to create a self-report scale that is gender-sensitive and includes internalised symptomatology. Although the scientific literature describes differential symptomatology between boys and girls with ADHD, no internationally validated instrument has addressed this specific dimension.

### 2.3. Questionnaire Design

To ascertain the opinions of the experts and reach a consensus, three online forms were created using Google Forms [53]. The forms were sent with a brief description of the project, the objectives, and the corresponding instructions. As they were online forms, they had the advantage that the expert professionals could access them from any personal device and at the time that best suited their schedule. Therefore, by allowing a reasonable response time, it was easy to collect and compile the responses.

The forms were designed in such a way that all questions had to be answered by all experts; they were mandatory. This ensured that there were no missed questions or omitted data, which would have complicated the analysis of the responses.

### 2.4. Data Collection

The first round of the e-Delphi method was launched electronically in May 2023, the second round in October 2023, and the third round in January 2024. The forms were organised coherently with the development of the scale, i.e., an orderly timeline was followed that accompanied the whole process of creating the scale. There were three rounds. The first, in which experts were asked to identify ADHD cases, was based on DSM-5-TR criteria and key symptomatology used for the early detection of ADHD, allowing them to be classified according to the differential symptomatology between boys and girls aged between 6 and 16 years.

The second round consisted of assessing the symptomatology identified in the first round that would help to specifically identify ADHD in girls. Each identified symptom was individually rated on a numerical scale from 1 to 4 (with 1 being not relevant at all and 4 being very relevant). In addition, experts in this round could suggest items that had not been previously considered.

Finally, the third round consisted of transforming the symptomatology into a broad pool of items, which were distributed in the three dimensions (inattention, hyperactivity/impulsivity, and “internalisation”). The items distributed in the three dimensions made up the first version of the scale, which was submitted for experts to deliver their opinions, where each item was re-evaluated based on whether it should be included or not in the final version and whether it is the most suitable format for the scale.

Finally, the final version of the scale was pilot-tested on a group of 20 children aged 6–12 years to assess the comprehension of each item and the appropriateness of the format.

### 2.5. Participants and Recruitment

To conduct this study, it was deemed essential to consult with leading health professionals involved in the field of mental health and who specialize in child care, both in the private and public sectors. Hence, a purposive sampling approach was undertaken.

Communication was established with experts possessing extensive experience in the primary topic of the study, centred around the approach and diagnosis of ADHD in childhood and adolescence. These professionals were selected based on their track record in child mental health, as well as their contributions and publications in mental health and gender studies. They were required to have a minimum of 10 years of clinical experience in the study’s field. Direct email invitations were sent to recruit the experts.

The professional panel comprised a total of 10 experts, including a neuro-paediatrician, two child psychiatrists, two clinical psychologists, two neuropsychologists (from both public and private sectors), a general health psychologist, a psychologist researcher in gender studies, and a nurse and researcher specializing in mental health. Regarding the sample size, the total number of professionals participating in this study fell within an acceptable range. The Rand Corporation, an international research and development organization, suggests that with a minimum of 7 experts, the margin of error decreases significantly for each additional expert, and it is not advisable to exceed 30 experts [52,53].

### 2.6. Data Analysis

With the expert panel set up and the three rounds of the e-Delphi method completed, the results of the e-Delphi method were coded and analysed descriptively as responses were generated, taking into account the general patterns of response and their frequency and percentages, indicating the convergence of opinions, general consensus, and the achievement of the objective.

## 3. Results

The final e-Delphi results were analysed to directly achieve the purpose of the study to obtain a gender-sensitive ADHD screening scale for boys and girls.

### 3.1. Round 1

Table 1 shows the frequency of inattention and hyperactivity/impulsivity symptoms assessed by the expert panel. Symptoms related to inattention had the highest frequency and agreement compared to the symptoms associated with hyperactivity.

The expert panel identified seven possible specific symptoms for the detection of ADHD in girls. These symptoms were grouped under the criterion “internalising”. Table 2 shows these symptoms.

### 3.2. Round 2

In this second round, the experts assessed the relevance of the symptomatology identified in the previous round as specific symptomatology for detecting ADHD in girls. Four of the seven symptoms generated were rated as relevant (3) or very relevant (4) by the expert panel (Table 3). No expert considered adding any additional symptoms to the proposal.

### 3.3. Round 3

Following the assessment of items in the previous rounds and considering all contributions from the panel of experts, the research team compiled a list of 62 items distributed across three dimensions. The first dimension focused on inattention with 21 items, the second on hyperactivity/impulsivity with 20 items, and the third on a differential symptomatology termed “internalisation” with 21 items.

Each expert evaluated every item to determine its relevance for inclusion in the scale. Out of the 21 items proposed for the inattention dimension, 10 received a full consensus from the experts for inclusion in the final scale. Similarly, for the hyperactivity/impulsivity dimension, 10 out of the 20 items proposed were agreed upon, and for the internalisation dimension, 11 out of 21 items gained expert consensus. Consequently, a final list of 31 items was established.

In the latter part of the third phase, the panel was asked whether they would propose additions or alterations to the scale’s content and format. Based on the expert input, three items were reformulated and an additional three items were included and deemed significant for identifying ADHD in girls.

Requests were also made regarding the standardization, adaptation, and expansion of the scale to include versions for parents, schools, and adults. It was suggested that in the final version, items should not be distributed by dimensions to prevent response selection bias.

The final scale comprised 34 items, with 11 items corresponding to the inattention dimension, 10 to the hyperactivity/impulsivity dimension, and 13 to the internalisation dimension. Each item on the scale is assessed using four possible responses: 1 for NEVER or ALMOST NEVER, 2 for FEW TIMES, 3 for MANY TIMES, and 4 for ALWAYS or ALMOST ALWAYS.

A summary table of the proposed scale is shown in Table 4 below.

The scale is presented in Appendix A (Model instructions: ADHD SCALE FOR BOYS AND GIRLS SELF-REPORT—6 to 16 years) and Appendix B (ADHD SCALE FOR BOYS AND GIRLS SELF-REPORT—6 to 16 years).

Finally, a pilot test was conducted with children to assess their comprehension of the items and the scale format. This pilot test involved a small sample of 20 Spanish-speaking boys and girls from different schools and towns in Catalonia aged between 6 and 16 years old, as older children are expected to encounter fewer difficulties in comprehending the items. For the pilot test, corresponding informed consent forms were signed.

During the review conducted by the children, no significant changes to the scale were deemed necessary, except for adjustments in vocabulary and the adoption of less formal and more accessible language. For instance, “I feel overwhelmed” was changed to “I find it difficult to control” and “to decide between two options” was modified to “to decide between two things”.

## 4. Discussion

This study aimed to develop an instrument used for the early detection of ADHD that considers the gender perspective and is sensitive to identifying girls with ADHD. The goal was to address a recognized need observed by many professionals in their daily clinical practice. Specialized professionals in neuropsychological assessment, learning difficulty evaluation, and child and adolescent clinical practice have noted for years that girls often do not fit neatly into certain diagnostic categories, yet their functional difficulties persist without adequate diagnosis and treatment.

Numerous studies over recent decades support gender differences in ADHD [14,15,16,17,18,19,20,21,22,23,24,25,26,27,28,29,30,31,32,33,34,35,36,37,38,39,40]. However, there is ongoing controversy regarding how to detect gender differences and their clinical manifestation in boys and girls. Studies analysing ADHD assessment based on the DSM criteria and its diagnosis report that DSM criteria analyses show symptom invariance, regardless of the gender of the children assessed [41,42,43]. However, some authors suggest that more comprehensive analyses using clinical population samples are necessary in order to assess whether symptom invariance holds under these conditions. Other studies question the adequacy of DSM criteria for detecting ADHD in girls [25].

Therefore, in this study, we initially consulted experts from various disciplines involved in ADHD to understand how gender manifests in girls and how this symptomatology can be addressed. The first two rounds yielded broad consensus on the most relevant symptomatology to consider. Both the inattention and hyperactivity/impulsivity dimensions, along with more internalized symptomatology, were grouped under the “internalisation” dimension.

Despite existing controversies, gender differences have been analysed and evidenced. For instance, significant interactions between gender diagnosis and anxiety symptomatology have been reported in girls [28,30,41], along with lower rates of externalizing behaviours and hyperactivity, as well as higher rates of internalizing symptomatology and inattention in girls [21,32]. Consistently, our study’s analysis of expert opinions regarding classic ADHD dimensions indicated that inattention symptomatology carries more weight for detecting ADHD in girls than hyperactivity/impulsivity, although the latter should still be included in the scale.

Regarding internalizing symptomatology in girls, some authors like Skogli [40] and his research team recommend integrating self-reports of internalizing symptomatology with ADHD rating and detection scales, given the lack of specific instruments included in this symptomatology for ADHD detection and diagnosis. Hence, the internalizing symptomatology identified and agreed upon by experts, as expressed in girls, is categorized under the dimension termed “internalisation”.

Another crucial aspect considered in this study is the assessment of ADHD. Historically, ADHD diagnosis has relied on various informants, particularly reports from third parties (primary caregivers, teachers, etc.), which are susceptible to multiple biases [51]; even professionals themselves can be subject to bias [54]. Guided by this understanding, the design of this scale has consistently prioritized the child’s own self-reporting, i.e., giving children a voice regarding their symptoms and avoiding instruments solely based on third-party behaviour observation. However, it is not precluded that in subsequent studies, this scale could be complemented with others by incorporating input from other informants.

Numerous studies have addressed ADHD assessment and detection instruments [55,56,57,58,59]. However, most instruments have focused on behavioural aspects reported by children or their informants rather than the children’s internal symptoms. Currently, several studies support that children can be reliable informants of their difficulties or they are deemed no less valid than third parties [60,61,62]. Thus, due to the more internal basis of differential symptomatology between boys and girls [63,64], the decision was made to develop a self-report scale for boys and girls aged 6 to 12 years. Moreover, the study demonstrated significant consensus among the expert panel regarding this internalizing symptomatology.

Given the differential presentation of ADHD according to gender, the early detection of this symptomatology in girls facilitates better disorder management, prevents long-term comorbidities, and reduces the risk of low self-esteem and/or suicidal behaviour in girls. It also lowers healthcare costs associated with inadequate or failed treatments [63,64]. Consequently, our research team opted for an early detection instrument capable of identifying ADHD-compatible symptoms in boys and sensitive to early detection in girls.

In light of this review, the development of ADHD screening instruments that consider the gender perspective and transcend differentiated normative groups was imperative; a factor that guided this study. In the literature review of ADHD assessment instruments according to gender, no validated scales were found. However, a scale design, exclusively for girls, the ADHD Self-Rating Scale for Girls (+Teenage Girls), developed by Patricia Quinn, Kathleen Nadeau, and Ellen Littman and published in “Understanding Girls with ADHD” in 2005, has not been validated or adapted at present.

The scale, composed of 34 items, with 11 corresponding to the inattention dimension, 10 to the hyperactivity/impulsivity dimension, and 13 to the “internalisation” dimension, was developed under the consensus of professionals with over 10 years of experience in the field. It adheres to standards established by the scientific literature on ADHD symptoms and gender, current diagnostic tools, and the reliability of minors’ reports.

The limitations of this study are mainly centred on the fact that the scale was developed in the Spanish context, with Spanish professionals, and in a specific language (Spanish). This does not favour the generalisation of results, but as a research team, we hope that the scale can eventually be adapted to English and other languages.

## 5. Conclusions

Using the e-Delphi method, developed to achieve consensus in health research, and involving experts with over a decade of clinical experience, a scale for Attention-Deficit Hyperactivity Disorder (ADHD) was crafted. This scale, grounded in a robust theory of ADHD symptomatology, is tailored to accommodate the symptomatic variations between boys and girls, aiming to mitigate gender bias in diagnosis. It encompasses both traditional dimensions of the disorder and internalized symptoms, addressing challenges in emotional self-regulation. The panel of experts exhibited a high level of concurrence throughout the Delphi process rounds, thus endorsing the content of the developed scale. This concise self-administered tool is designed for children aged 6 to 16 years and represents a significant stride towards crafting gender-neutral psychological instruments for ADHD. However, further research is warranted in order to assess its psychometric properties, as the scale requires an exhaustive study of its reliability and validity.

## Figures and Tables

**Table 1:** Frequency of expert responses to the DSM-5-TR items for the ADHD screening scale for boys and girls.

Inattention	*n*	%
Often does not pay sufficient attention to detail or makes careless mistakes in schoolwork, work, or other activities.	10	100%
Often has difficulty sustaining attention in tasks or play activities.	9	90%
Often does not seem to listen when spoken to directly.	9	90%
Often does not follow instructions and does not complete school work, assignments, or duties at the workplace.	9	90%
Often has difficulties in organising tasks and activities.	10	10%
Often avoids, dislikes, or is reluctant to engage in tasks that require sustained mental effort.	8	8%
Often misplaces objects needed for tasks or activities.	7	70%
Often easily distracted by irrelevant stimuli.	9	90%
Often neglected in daily activities.	8	80%
Often experiences hyperactivity/impulsivity.	***n***	**%**
Often moves hands or feet excessively or shifts in seat.	3	30%
Often leaves his or her seat in class or in other situations where he or she is expected to remain seated.	1	10%
Often runs or jumps excessively in situations where it is inappropriate to do so.	1	10%
Often has difficulty playing or engaging in leisure activities.	1	10%
Often “on the move” or acts as if he or she has an engine.	1	10%
Often talks too much.	5	50%
Often precipitates answers before questions are completed.	3	30%
Often has difficulty in keeping his turn to speak.	3	30%
Often interrupts or intrudes on the activities of others.	3	30%

**Table 2:** Proposed symptomatology of the expert panel.

Often scattered in conversations. Often shows anger for no reason. Often has explosion in their responses. Often has sudden mood swings. Often their efforts are not reflected. Often struggles to make decisions. Often has rumination and high flow of thoughts.

**Table 3:** Relevance of the symptomatology assessed by the panel of experts.

	Not or Hardly Relevant		Relevant or Very Relevant	
	N	%	*n*	%
Often scattered in conversations.	0	0%	10	100%
Often shows anger for no reason.	1	10%	9	90%
Often explodes in their responses.	0	0%	10	100%
Often has sudden mood swings.	3	30%	7	70%
Often does not see his or her efforts reflected.	2	20%	8	80%
Often struggles to make decisions.	3	30%	7	70%
Often has rumination and a high flow of thoughts.	2	20%	8	80%

**Table 4:** Attention-Deficit Hyperactivity Disorder screening scale for boys and girls.

Application: Individual and collective in self-report format.
Time: Variable, from 30 to 40 min.
Age: From 6 to 16 years old.
Purpose: To design an instrument for the early detection of symptoms associated with Attention-Deficit Hyperactivity Disorder for boys that is also sensitive to the detection of girls (based on the exploration of three dimensions: one of inattention, one of hyperactivity/impulsivity, and a third of internalisation).

## Data Availability

The data that support the findings of this study are available from the corresponding author upon reasonable request.
